# Supplementary material for: A New Type of Nonsuppressible Viremia Produced by HIV-Infected Macrophage
Source: bioRxiv. 2025 Sep 3:2025.09.02.673877. Preprint. [Version 1] doi: 10.1101/2025.09.02.673877 (PMC12424842; doi:10.1101/2025.09.02.673877)
Supplement: Supplement 1 — Supplemental Table 1: P1 Prior treatment history [file media-1.pdf]

Supplemental Table 1: P1 Prior treatment history

Regimen	Start Year	End Year	Years on regimen
AZT/3TC/EFV	2004	2005	1.3
FTC/TAF/ATV/r	2009	2010	1.2
FTC/TDF/ATV/r	2010	2013	~3
EVG/COBI/FTC/TDF	2014	2016	~2
BIC/FTC/TAF	2018	2018	~1
