# Supplementary material for: A New Type of Nonsuppressible Viremia Produced by HIV-Infected Macrophage
Source: bioRxiv. 2025 Sep 3:2025.09.02.673877. Preprint. [Version 1] doi: 10.1101/2025.09.02.673877 (PMC12424842; doi:10.1101/2025.09.02.673877)
Supplement: Supplement 2 — Supplemental Table 2: Primers [file media-2.pdf]

Supplemental Table 2: Primers

Region	Primer Type	Primer name	Sequence
PR HXB2# 2164-2591	cDNA	R2614_PID	GTGACTGGAGTTCAGACGTGTGCTCTTCCGATCTNNNNNNNNNCAG TTTAACTTTTGGGCCATCCATTCC
	PCR1 F	F2163AD	GCCTCCCTCGCGCCATCAGAGATGTGTATAAGAGACAGNNNNTCAG AGCAGACCAGAGCCAACAGCCCCA
RT HXB2# 2648-3257	cDNA	R3284_PID11	GTGACTGGAGTTCAGACGTGTGCTCTTCCGATCTNNNNNNNNNNNC AGTCACTATAGGCTGTACTGTCCATTTATC
	PCR1 F	F2620_AD	GCCTCCCTCGCGCCATCAGAGATGTGTATAAGAGACAGNNNNGGCC ATTGACAGAAGAAAAAATAAAAGC
INT HXB2# 4384-4751	cDNA	R4752_PID11	GTGACTGGAGTTCAGACGTGTGCTCTTCCGATCTNNNNNNNNNNNA TCGAATACTGCCATTTGTACTGC
	PCR1 F	F4383_AD	GCCTCCCTCGCGCCATCAGAGATGTGTATAAGAGACAGNNNNAAAA GGAGAAGCCATGCATG
V1V3 HXB2# 6585-7208	cDNA	R7209_PID11	GTGACTGGAGTTCAGACGTGTGCTCTTCCGATCTNNNNNNNNNNNC AGTCCATTTTGCTYTAYTRABVTTACAATRTGC
	PCR1 F	V1F_AD	GCCTCCCTCGCGCCATCAGAGATGTGTATAAGAGACAGNNNNTTAT GGGATCAAAGCCTAAAGCCATGTGTA
VPR HXB2# 5466-5961	cDNA	R5962_PID	GTGACTGGAGTTCAGACGTGTGCTCTTCCGATCTNNNNNNNNNNNC AGTGCTTCTTCCTGCCATAGGAGATG
	PCR1 F	F5488_AD	GCCTCCCTCGCGCCATCAGAGATGTGTATAAGAGACAGNNNNTAGG ATCTCTACAGTACTTGGCAC
NFL	F	U5B1F	CCTTGAGTGCTTCAAGTAGTGTGTGCCCGTCTGT
	R	R3B3R	ACTACTTGAAGCACTCAAGGCAAGCTTTATTG
FLIP-seq PCR1	F	BLOuterF	AAATCTCTAGCAGTGGCGCCCGAACAG
	R	BLOuterR	TGAGGGATCTCTAGTTACCAGAGTC
FLIP-seq PCR2	F	U5-638F Guin	GCGCCCGAACAGGGACYTGAAARCGAAAG
	R	NFL RP BE	GCACTCAAGGCAAGCTTTATTGAGGCTTA
3’HG	F	4653F	CCCTACAATCCCCAAAGTCAAGGAG
	R	Ofm19	GCACTCAAGGCAAGCTTTATTGAGGCTTA
5’HG	F	LTRgagF	TCTCGACGCAGGACTCG
	R	PB5HChaviR	CTTGCCACACAATCATCACCTGCCAT
Env Cloning	F	B5957F-TOPO	CACCTTAGGCATCTCCTATGGCAGGAAGAAG
