# Supplementary material for: A New Type of Nonsuppressible Viremia Produced by HIV-Infected Macrophage
Source: bioRxiv. 2025 Sep 3:2025.09.02.673877. Preprint. [Version 1] doi: 10.1101/2025.09.02.673877 (PMC12424842; doi:10.1101/2025.09.02.673877)
Supplement: Supplement 3 [file media-3.pdf]

	R	B8904R-TOPO	GTCTCGAGATACTGCTCCCACCC
ddPCR	HIV ddPCR	LTRgagF	TCTCGACGCAGGACTCG
		LTRgagR	TACTGACGCTCTCGCACC
		LTRgag_Probe	CTCTCTCCTTCTAGCCTC
	RPP30 ddpCR	RPP30F	GATTTGGACCTGCGAGCG
		RPP30R	GCGGCTGTCTCCACAAGT
		RPP30_Probe	CTGACCTGAAGGCTCT
