# Supplementary material for: A New Type of Nonsuppressible Viremia Produced by HIV-Infected Macrophage
Source: bioRxiv. 2025 Sep 3:2025.09.02.673877. Preprint. [Version 1] doi: 10.1101/2025.09.02.673877 (PMC12424842; doi:10.1101/2025.09.02.673877)
Supplement: Supplement 4 — Supplemental Table 3: Limiting dilution sequencing methods [file media-4.pdf]

Supplemental Table 3: List of limiting dilution sequencing methods

Participant	Timepoint	Sample Type	Sequencing Amplicon	Sequencing Method
Participant 1	-165 wpa	Plasma RNA	3'HG and NFL	PacBio, MGH
	0 wpa	Plasma RNA	3'HG and NFL	PacBio, MGH, Plasmidsaurus
	8 wpa	Plasma RNA	3'HG and NFL	PacBio, MGH
	32 wpa	Proviral DNA	3'HG and NFL	FLIP-seq, PacBio, Plasmidsaurs
Participant 2	-2 wpa	Plasma RNA	Full *env*	Sangar
	8 wpa	Plasma RNA	Full *env*	Sangar
	19 wpa	Plasma RNA	Full *env*	Sangar
Participant 3	21 wpa	Plasma RNA	3'HG and NFL	PacBio, Plasmidsaurs
	34 wpa	Plasma RNA	3'HG and NFL	PacBio, Plasmidsaurs
	58 wpa	Proviral DNA	3'HG	PacBio, Plasmidsaurs
Participant 4	58 wpa	Plasma RNA	3'HG	MGH, Plasmidsaurus
	75 wpa	Proviral DNA	3'HG	Plasmidsaurus
