# Supplementary material for: A New Type of Nonsuppressible Viremia Produced by HIV-Infected Macrophage
Source: bioRxiv. 2025 Sep 3:2025.09.02.673877. Preprint. [Version 1] doi: 10.1101/2025.09.02.673877 (PMC12424842; doi:10.1101/2025.09.02.673877)
Supplement: Supplement 5 — Supplemental Figure 1: Detailed treatment histories for P3 and P4. (A-B) Viral loads (HIV-1 RNA cp/mL) and CD4+ T cell in cells/μL for P3 and P4. Limits of quantification for viral load tests are marked as a black, dashed lines. Timepoints when plasma was sampled and used for viral RNA sequencing are represented by circles (⚫) and PBMC samples are represented by squares (▄). Colors indicate when each sample was collected. Phylogenetic trees of partial HIV-1 env (V1V3) sequences (identical sequences collapsed) are shown and slow decay lineages are marked with stars. [file media-5.pdf]

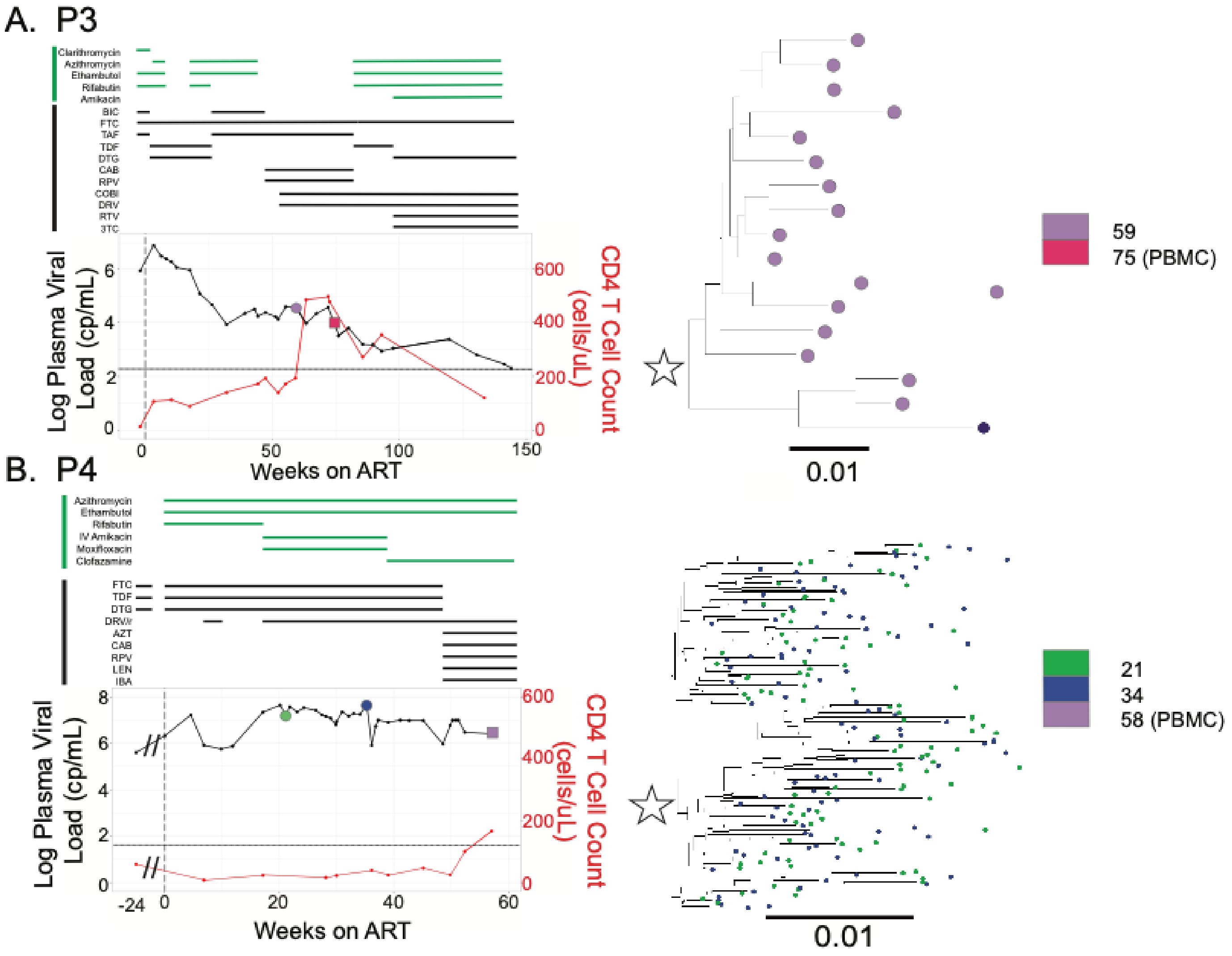


**Supplemental Figure 1: Detailed treatment histories for P3 and P4.** (A-B) Viral loads (HIV-1 RNA cp/mL) and CD4+ T cell in cells/µL for P3 and P4. Limits of quantification for viral load tests are marked as a black, dashed lines. Timepoints when plasma was sampled and used for viral RNA sequencing are represented by circles (●) and PBMC samples are represented by squares (■). Colors indicate when each sample was collected. Phylogenetic trees of partial HIV-1 *env* (V1V3) sequences (identical sequences collapsed) are shown and slow decay lineages are marked with stars.
