# Supplementary material for: A New Type of Nonsuppressible Viremia Produced by HIV-Infected Macrophage
Source: bioRxiv. 2025 Sep 3:2025.09.02.673877. Preprint. [Version 1] doi: 10.1101/2025.09.02.673877 (PMC12424842; doi:10.1101/2025.09.02.673877)
Supplement: Supplement 6 — Supplement Figure 2: Phylogenetic tree analyzing pre-ART HIV-1 RNA sequences from P1. Phylogenetic tree of partial HIV-1 env (V1V3) sequences (identical sequences collapsed) of viral RNA in plasma collected at 2 timepoints before ART initiation (see timepoints and colors in 1A) and the lineage that persists after ART is noted with a star. [file media-6.pdf]

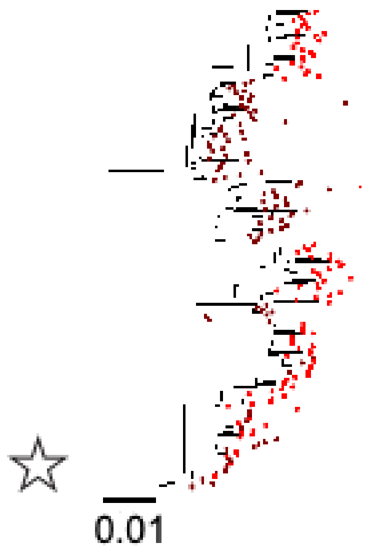

Supplement Figure 2: Phylogenetic tree analyzing pre-ART HIV-1 RNA sequences from Participant 1. Phylogenetic tree of partial HIV-1 *env* (V1V3) sequences (identical sequences collapsed) of viral RNA in plasma collected at 2 timepoints before ART initiation (see timepoints and colors in 1A) and the lineage that persists after ART is noted with a star.
