# Supplementary material for: A New Type of Nonsuppressible Viremia Produced by HIV-Infected Macrophage
Source: bioRxiv. 2025 Sep 3:2025.09.02.673877. Preprint. [Version 1] doi: 10.1101/2025.09.02.673877 (PMC12424842; doi:10.1101/2025.09.02.673877)
Supplement: Supplement 8 — Supplement Figure 4: Proportion of vpr defects within the entire defective vpr gene population at each sampled timepoint. Proportions determined by Miseq Primer ID sequencing. [file media-8.pdf]

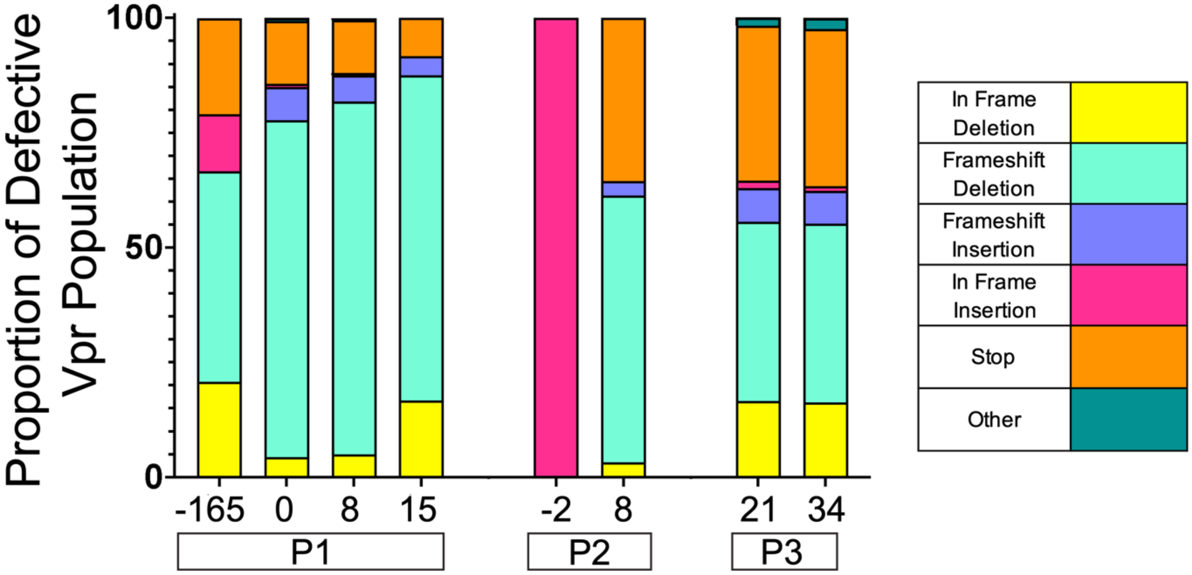

Supplement Figure 4: Proportion of *vpr* defects within the entire defective *vpr* gene population at each sampled timepoint. Proportions determined by Miseq Primer ID sequencing.
